# Multiple Myeloma Measurable Residual Disease: Spotting Three Different Detection Methods

**DOI:** 10.3390/ijms27219447

**Published:** 2026-10-23

**Authors:** Sara Tartaglione, Marzia Tagliaferro, Maria Cristina Scerpa, Valerio Basile, Francesca D’Ambrosio, Patrizia Natali, Mariapaola Marino, Francesco Equitani, Luca Maurillo, Umberto Basile

**Affiliations:** 1 Unit of Clinical Pathology, Santa Maria Goretti Hospital, 04100 Latina, Italym.tagliaferro@ausl.latina.it (M.T.);; 2 Unità Operativa Complessa Ematologia con Trapianto, Santa Maria Goretti Hospital, 04100 Latina, Italy; 3 Clinical Pathology Unit and Cancer Biobank, Department of Research and Advanced Technologies, Regina Elena National Cancer Institute—Istituto di Ricovero e Cura a Carattere Scientifico (I.R.C.C.S.), 00144 Rome, Italy; 4 Struttura Complessa Interaziendale Laboratorio Analisi Chimico Cliniche e Microbiologiche—Azienda Ospedaliera Santa Croce e Carle, 12100 Cuneo, Italy; 5 Dipartimento di Medicina e Chirurgia Traslazionale, Sezione di Patologia Generale, Università Cattolica del Sacro Cuore, 00168 Rome, Italy; 6 Fondazione Policlinico Universitario “A. Gemelli”—Istituto di Ricovero e Cura a Carattere Scientifico (I.R.C.C.S.), 00168 Rome, Italy; 7 Unità Operativa Complessa-Medicina Trasfusionale, Santa Maria Goretti Hospital, 04100 Latina, Italy; 8 Dipartimento di Medicina Traslazionale e Medicina di Precisione, La Sapienza Università di Roma, 00185 Rome, Italy; 9 Unità Operativa Complessa-Ematologia, Ospedale S. Maria Goretti, 04100 Latina, Italy

**Keywords:** multiple myeloma, measurable residual disease, next-generation flow cytometry, next-generation sequencing, mass spectrometry, monoclonal protein, free light chains, bone marrow, peripheral blood monitoring, prognosis, risk stratification

## Abstract

Multiple myeloma (MM) is the second most common hematologic malignancy. It is still largely incurable, but treatment has changed greatly with proteasome inhibitors, immunomodulatory drugs, monoclonal and bispecific antibodies, autologous stem cell transplantation and chimeric antigen receptor T cells. Most patients now achieve a complete response. As a result, conventional criteria, based on monoclonal protein, serum free light chains and bone marrow plasma cell counts, can no longer distinguish different depths of response. Measurable residual disease (MRD) has therefore become the reference endpoint. The International Myeloma Working Group defines MRD negativity as the absence of clonal plasma cells among at least 100,000 bone marrow cells. MRD negativity predicts progression-free and overall survival, independently of cytogenetic risk and disease stage. The US Food and Drug Administration has also accepted it as an early endpoint for accelerated approval. This review examines the three main technologies now used to assess MRD in MM. Next-generation flow cytometry is standardized through the EuroFlow two-tube, eight-color protocol. It gives same-day results and can be applied to all patients without a baseline sample. Next-generation sequencing of clonal immunoglobulin gene rearrangements is more sensitive and can be used on stored samples. It can also follow clonal evolution and the appearance of resistance mutations. Mass spectrometry is performed on peripheral blood. It detects the patient-specific clonotypic immunoglobulin with much higher sensitivity than electrophoretic methods and allows frequent, minimally invasive monitoring. For now, however, it complements marrow-based testing and does not replace it. For each method, we discuss analytical principles, sensitivity, standardization, pre-analytical requirements, clinical validation and limitations, and we briefly consider the growing contribution of artificial intelligence. The review also covers the role of cytogenetics and fluorescence in situ hybridization in baseline risk assessment. Finally, we discuss the limits of MRD testing in a disease that is often unevenly distributed between marrow, extramedullary sites and peripheral blood. Current evidence suggests that combining marrow-based and blood-based assays may identify residual disease most accurately. This combined approach has not yet been validated prospectively, and for now it should be used within clinical trials.

## 1. Introduction

Multiple myeloma (MM) is the second most common hematologic malignancy (~10%) and is defined by uncontrolled clonal plasma cell proliferation within the bone marrow (BM) with monoclonal protein (M-protein) in serum or urine [1,2].

This disease is characterized by a wide spectrum of clinical signs and symptoms such as anemia, hypercalcemia, renal dysfunction, immunodeficiency, and bone lesions. Three subtypes of MM are recognized according to the monoclonal protein secreted by the tumor: intact immunoglobulin (Ig) myeloma, light-chain-only myeloma, and non-secretory myeloma. Intact Ig myeloma accounts for 80% of all myelomas and 89% of these cases also secrete an excess of free light chains (FLCs) resulting in an abnormal kappa/lambda (κ/λ) ratio at diagnosis. Kappa and lambda FLCs are low-molecular-weight proteins produced in slight excess during immunoglobulin synthesis and released into the bloodstream. Over 99% of circulating FLCs are filtered, reabsorbed, and metabolized by the kidneys, so their blood levels depend on the balance between production and renal clearance [3].

Because of their short half-life (2–6 h), in subjects with normal kidney function their serum levels can be considered a direct marker of B-cell activity: the quantitative assay of κ and λ FLCs and the κ/λ ratio has become a useful diagnostic tool in plasma cell dyscrasias and autoimmune diseases [4,5,6,7].

**Figure 1:**
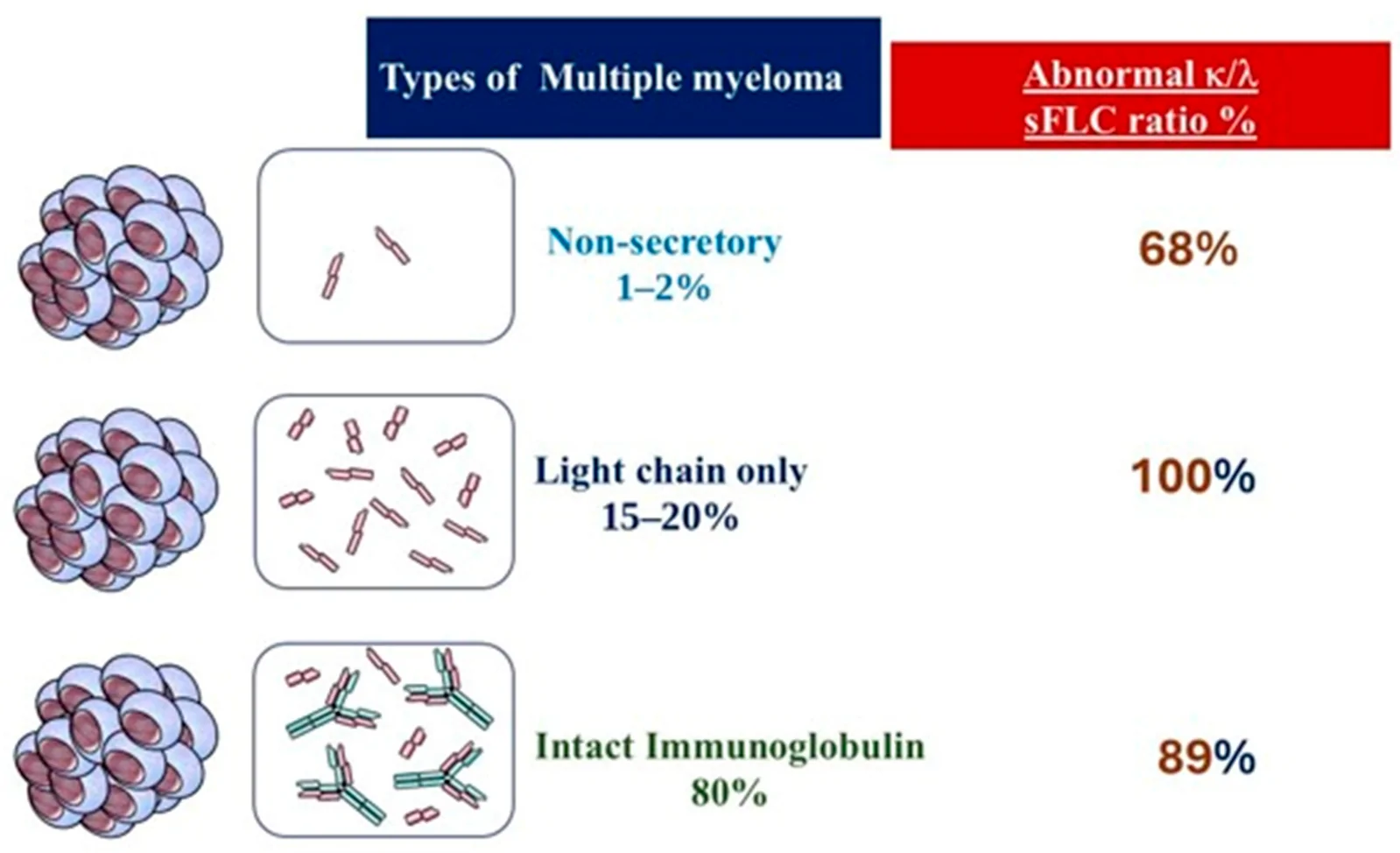
Secretory subtypes of malignant plasma cells and κ/λ ratio at diagnosis.

Light-chain-only myeloma accounts for 15–20% of all myelomas: all cases secrete excess FLCs and consequently show an altered κ/λ ratio at diagnosis. A small proportion of cases are classified as non-secretory myeloma and were traditionally thought not to secrete any paraprotein. However, at least 68% of these cases (and an even higher proportion in some institutions) secrete excess FLCs and therefore present an abnormal ratio at diagnosis (Figure 1) [8].

In MM, plasma cell clones with different characteristics and genetic abnormalities have been identified and extensively studied. Many clones—approximately half of all cases detected at relapse—show genetic features that differ from those observed at initial diagnosis [9].

**Figure 2:**
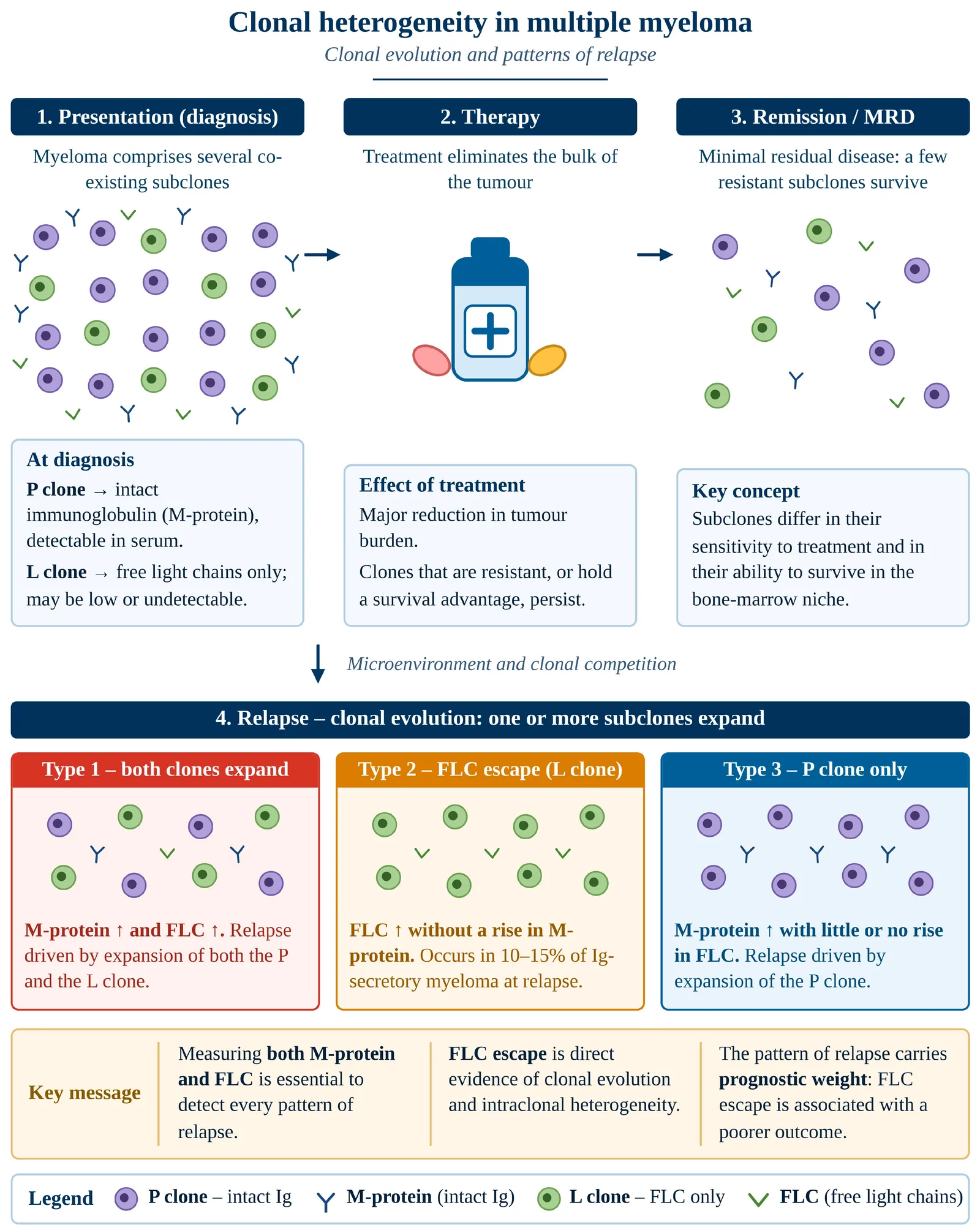
Clonal evolution and relapse patterns in MM: Relapse can be characterized by both free-light-chains and intact-immunoglobulin-secreting plasma cells (type 1); by free-light-chains-secreting plasma cells only (type 2); or by intact-immunoglobulin-secreting plasma cells only (type 3). Arrows indicate the sequence of disease phases (diagnosis, therapy, remission/MRD and relapse). In the boxes, → links each clone to the protein it secretes, and ↑ indicates an increase in the serum level of the corresponding protein. Over the past decade, new treatments have substantially improved survival in MM. Proteasome inhibitors, immunomodulatory drugs, autologous stem cell transplantation (ASCT), bispecific antibodies, chimeric antigen receptor (CAR) T cell therapy and monoclonal antibodies have all contributed [2,11]. Monoclonal antibodies act directly on their target cells, but they may also have wider immunomodulatory effects through interactions between different immune-cell compartments [12,13]. At present, B-cell maturation antigen (BCMA) is the most successful target for CAR-T cell therapy in MM. Results in relapsed/refractory MM (RRMM) are encouraging. However, the benefit of anti-BCMA CAR-T cells is often short-lived. Interactions between CAR-T cells, tumor cells and a complex tumor microenvironment lead to antigen escape and CAR-T cell exhaustion [14].

Light chain escape (LCE) was among the first clinical manifestations of clonal heterogeneity to be described: a clone produces a complete antibody, while another secretes only an FLC. Chemotherapy acts differentially against the different clones, as do other evolutionary pressures such as the microenvironment or competition for the stem cell niche. The selective pressures applied determine which clone(s) survive and give rise to relapse. The different clonal composition at relapse ultimately affects sensitivities to subsequent treatments and therefore survival, as shown in Figure 2 [10]. How the three MRD techniques discussed in this review behave in each of these relapse patterns is considered in Section 7.

With currently available regimens, transplant-eligible and transplant-ineligible patients with newly diagnosed MM (NDMM) generally achieve a first progression-free survival (PFS) of more than 10 years and 5 years, respectively. However, prognosis varies widely and depends mainly on disease biology and on response to treatment. Therefore, regular disease monitoring is essential, and reliable markers are needed to define prognosis and guide therapy [15].

## 2. The Role of Measurable Residual Disease

Measurable residual disease (MRD) positivity is defined by the International Myeloma Working Group (IMWG) as the persistence of a very small number of malignant plasma cells during or after therapy. Recent guidelines consider MRD a more sensitive and clinically meaningful response criterion than traditional endpoints, such as complete response (CR) and stringent CR [16].

In 2024, the US Food and Drug Administration (FDA) endorsed MRD negativity as an early endpoint for the accelerated approval of new MM treatments. In 2026, the FDA issued a draft guidance for industry along the same lines. It recommends MRD and CR as primary endpoints to support accelerated approval of drugs and biologics in MM clinical trials [17,18].

According to IMWG criteria, undetectable MRD is defined as the absence of detectable myeloma cells among ≥ 100,000 bone marrow cells (i.e., with an assay reaching a limit of detection of at least 10^−5^) [16].

Three terms are often used interchangeably in the MRD literature, but they mean different things. We have tried to keep them apart in this review. The limit of detection (LOD) is the lowest level of residual disease that an assay can reliably distinguish from zero, while the limit of quantification (LOQ) is the lowest level that can be measured with acceptable precision and accuracy. Both are analytical properties. In flow- and sequencing-based assays, they depend directly on the number of cells, or the amount of DNA, analyzed in each sample. Clinical sensitivity is a different concept. It describes how well a negative or positive result identifies the patients who will or will not relapse. It depends on sampling, on how the disease is distributed in the body and on its biology, as much as on the analytical performance of the test [16].

The prognostic role of MRD is now well established. Deeper remission, shown by the absence of MRD at levels <1 × 10^−5^ correlates with longer PFS and overall survival (OS), regardless of cytogenetic risk or disease stage. For patients with NDMM, the goal of treatment should therefore be the deepest possible remission [19,20,21,22,23]. Moreover, sustained MRD negativity over time leads to significantly longer PFS, regardless of the treatment received [24]. The prognostic value of MRD, however, should not be confused with its use to guide individual treatment decisions, which is still under investigation; we return to this distinction in the Discussion.

EHA-EMN guidelines recommend MRD testing from bone marrow aspirates using either next-generation flow (NGF) or next-generation sequencing (NGS) [25].

Assay sensitivity is a key determinant of the prognostic value of undetectable MRD. Better survival has been observed at 10^−6^ than at 10^−5^, especially in high-risk patients. Undetectable MRD with an assay reaching an LOD of 10^−7^ may predict PFS even better than 10^−5^ or 10^−6^. Testing at such high sensitivity, however, is technically demanding and time-consuming, and is often difficult outside clinical trials [15,26].

Clinical trials have reported good overall concordance between NGS and NGF for MRD assessment. They have also shown a strong correlation between deep, durable undetectable MRD and longer survival in patients with MM [27]. NGF is a two-tube, eight-color, antibody-based flow cytometry assay. It identifies MM plasma cells (PCs) with greater sensitivity than conventional flow cytometry, with an LOD of 2 × 10^−6^ (20 aberrant plasma cells per 10 million cells acquired) and an LOQ of 5 × 10^−6^ (50 aberrant plasma cells per 10 million cells acquired) [28]. NGS is a molecular method that detects clonal immunoglobulin gene rearrangements, with an LOD of about 10^−6^ [26].

Several randomized clinical trials have compared the two methods and found similar performance. The methods differ, however, in availability and in some pre-analytical and analytical features. NGF is widely applicable and standardized through EuroFlow protocols, while NGS provides reproducible results across centers [29].

NGF and NGS are currently the two reference techniques. BM-based MRD testing, however, has clear limitations. BM aspiration is unpleasant and costly, and hemodilution may affect sample quality. Marrow infiltration in MM is often patchy, which can lead to false-negative results. Finally, BM-based testing cannot detect extramedullary disease [30].

Negativity on both molecular testing and imaging has been shown to define the deepest category of response. For imaging, the most recent EHA-EMN guidelines recommend positron emission tomography/computed tomography (PET/CT) and diffusion-weighted magnetic resonance imaging (MRI). No gold standard blood-based method for MRD negativity exists yet [31].

Several blood-based approaches are under investigation, such as circulating cell-free DNA (cfDNA) and single-cell RNA sequencing. Among them, mass spectrometry (MS) is one of the most promising, because it has shown higher sensitivity than other blood-based methods. At present, however, it complements marrow-based testing and does not replace it. MS can detect low levels of M-protein by recognizing the sequence of the antigen-binding region (complementarity-determining region, CDR), which is unique to each plasma cell clone. Its specificity is also higher than that of serum immunofixation and electrophoresis, as confirmed in both clinical practice and clinical trials [32,33].

The aim of this review is to explore the role of these three different technologies (NGF, NGS and MS) in assessing MRD in patients with MM. We also discuss how MRD results should be read together with baseline cytogenetic and genomic risk, and what MRD testing cannot capture in a disease that is often spatially heterogeneous. Several reviews on MRD in MM have been published recently. Among them, Chen et al. described multiparametric flow cytometry, allele-specific oligonucleotide PCR, NGS and imaging, with liquid biopsy and MS mentioned briefly among future perspectives [34]. The present review is written from the standpoint of laboratory medicine and has a different focus. MS is treated here as one of the three main techniques, alongside NGF and NGS. We start from the biology of the M-protein and of clonal evolution, and use it to explain how each technique behaves in the different relapse patterns. We keep analytical performance (LOD, LOQ) separate from clinical sensitivity. We distinguish clonotype-based NGS for MRD from genomic profiling, and relate both to baseline cytogenetic risk. We compare NGF, NGS and MS using the same analytical, pre-analytical and regulatory criteria. Finally, we discuss how discordant results between marrow, blood and imaging should be interpreted.

## 3. Literature Search Strategy

This is a narrative review. We searched PubMed/MEDLINE, Scopus and Web of Science for articles published in English between January 2014 and May 2026, combining the following terms: “multiple myeloma”, “measurable residual disease”, “minimal residual disease”, “next-generation flow”, “next-generation sequencing”, “clonoSEQ”, “mass spectrometry”, “MALDI-TOF”, “clonotypic peptide”, “peripheral blood”, “circulating tumor cells”, “cell-free DNA”, “cytogenetics”, “FISH” and “artificial intelligence”. The reference lists of the retrieved papers were also screened by hand, and older landmark studies were added when they were needed for context. Priority was given to consensus statements and guidelines (IMWG, EHA-EMN and FDA guidance documents), to prospective trials and large retrospective series, and to analytical validation studies. Case reports and papers not dealing specifically with plasma cell disorders were excluded; a few recent congress abstracts were kept when they reported prospective comparisons not yet available as full papers. Since our aim was a critical overview rather than a pooled estimate, no formal systematic review or meta-analysis was carried out.

## 4. Next-Generation Flow Method

In 2016, the IMWG approved the current definition of MRD criteria, including MRD negativity as determined by flow cytometry [16].

Flow-MRD negativity is defined by multiparameter flow cytometry (MFC) with an LOD of at least 1 × 10^−5^ cells. Equivalent methods can also be used, such as EuroFlow-NGF. This is a highly sensitive (LOD 2 × 10^−6^) and standardized approach that uses two eight-color antibody tubes. EuroFlow-NGF employs ammonium chloride-based bulk lysis, followed by surface staining with antibodies against CD138-BV421, CD27-BV510, CD38 multiepitope (ME)-FITC, CD56-PE, CD45-PerCP-Cy5.5, CD19-PECy7, CD117-APC, and CD81-APC-C750 in Tube 1. In Tube 2, surface and intracellular staining is performed with antibodies against CD138-BV421, CD27- BV510, CD38 ME-FITC, CD56-PE, CD45-PerCP Cy5.5, CD19-PECy7, cytoplasmic (cy) Igκ-APC, and cyIgλ- APC-C750 after permeabilization [28,35]. This protocol has been validated for MRD assessment in several studies. Together with dedicated standard operating procedures, it is considered the worldwide gold standard [36].

In 2021, Yoroidaka et al. [37] compared the EuroFlow method with the DuraClone RE PC kit (DuraClone panel). This is a recently developed eight-color, single-tube dry antibody panel, analyzed with Kaluza software (version 1.5a, Beckman Coulter, Brea, CA, USA) for identification of aberrant plasma cells (aPCs) and MRD detection with an LOD of 1 × 10^−5^. The DuraClone panel differed from EuroFlow-NGF in the total number of cells acquired, but it gave similar percentages of total and normal PCs and similar MRD levels [37]. This comparison, however, does not prove that the two methods are equivalent. DuraClone RE PC is a surface-only panel: it contains no cytoplasmic κ/λ staining, so clonality cannot be confirmed by light-chain restriction and aberrant plasma cells have to be recognized from their immunophenotype alone. This is a real limitation when residual cells look much like normal plasma cells. The same applies when treatment, including anti-CD38 antibodies, has changed antigen expression. The two-tube EuroFlow design, with a multiepitope CD38 reagent and cytoplasmic light chains in the second tube, copes better with both situations. Fewer cells were also acquired with the single-tube kit, and its sensitivity was about five times lower (LOD 10^−5^ versus 2 × 10^−6^ for EuroFlow-NGF). In our view, DuraClone is a reasonable option for MRD assessment at the 10^−5^ level in laboratories where EuroFlow is not available. It should not, however, be considered equivalent to EuroFlow-NGF until it has been tested in larger series and against clinical outcomes [28,37].

The main advantages of NGF are its wide applicability, feasibility even without a baseline diagnostic sample, and rapid turnaround time (3–4 h). Its main drawback is the need for fresh samples. Even so, it is faster and less expensive than NGS for MRD detection [19,38,39]. Previous studies have shown excellent concordance rates (often above 85%) between NGF and NGS when applied to the same BM sample at the standard sensitivity of 10^−5^. Both methods were highly predictive of long-term outcomes, particularly in patients with newly diagnosed MM [40,41]. A recent Japanese prospective study confirmed these findings. It found a strong correlation between MRD levels measured by NGF and NGS, and a significant association between MRD negativity and longer PFS [42].

Discordance between NGF and NGS is rare. When it occurs, NGS tends to be slightly more sensitive than standard NGF in some cytogenetic subgroups, such as the t(11;14) translocation, although published data are not entirely consistent [43,44]. Overall, MRD levels measured by NGF and NGS correlate strongly, and the two techniques may play complementary roles in monitoring MM [45].

NGF still has some pitfalls. These include hemodilution and phenotypic changes induced by therapy, for example by anti-CD38 monoclonal antibodies. Advanced gating strategies (e.g., NGF supported by Infinicyt™ software 2.0.6. (Cytognos, Salamanca, Spain) and multiepitope antibodies) and a high level of technical expertise are essential for reliable results [46].

In 2026, a multicenter Italian study compared the diagnostic performance and clinical utility of NGF and NGS. It found that NGF could overcome the problem of hemodilution. In addition, a prospective comparison within the phase III EMN24-ISKIA trial showed that NGF performance is not affected by disease phase, conventional response status or prior exposure to anti-CD38 mAbs [47,48].

NGF for MRD assessment in MM is currently based on BM sampling. However, a BM sample may not reflect the true extent of infiltration. It also misses extramedullary disease and some treatment effects in previously treated patients. For this reason, NGF has also been applied to peripheral blood to count circulating tumor cells (CTCs). CTC assessment has been widely shown to be reliable in patients with MM, both at diagnosis and during treatment [49].

Approximately 3–14 mL of blood are required to obtain ≥10^7^ cells per sample, sufficient to achieve adequate NGF sensitivity [50]. Because NGF detects CTCs easily, it may also help clarify how MM develops and spreads [51].

An MRD study applying NGF to peripheral blood from patients with MM has attempted to establish a cut-off value defining negativity for circulating pathological plasma cells [52].

Pre-enrichment strategies to further enhance NGF sensitivity have also been tested. One of them is density gradient medium (DGM) separation, which allows a much larger number of white blood cells to be processed (>60 × 10^6^ vs. 20 × 10^6^ with standard bulk lysis), achieving an LOD of 5.2 × 10^−7^. This is better than standard EuroFlow protocols, and processing time and cost are also reduced [53].

A new ultrasensitive EuroFlow-based approach, called “Bloodflow”, combines immunomagnetic enrichment of CD138+ circulating plasma cells in peripheral blood with NGF. It detects MRD better than conventional flow cytometry, reaching an LOD of up to 10^−8^. It also confirms positivity in samples in which standard methods are at their limit of detection, thereby allowing better, minimally invasive monitoring of patients with MM [54,55]. Bloodflow, however, is still a research method. The data published so far come from a few expert laboratories. The enrichment step adds cost and complexity. The procedure has not been standardized across centers and is not included in the IMWG response criteria. We also do not know yet whether a positive result at such low levels should lead to any change in treatment. Until prospective multicenter studies compare it with BM-based MRD and with clinical outcomes, Bloodflow belongs to research protocols rather than to clinical practice [55].

Based on recent studies and current clinical practice, NGF can be regarded as a standard, IMWG-endorsed method for MRD assessment in MM. It combines high sensitivity with standardization across centers. Undetectable MRD by NGF is one of the strongest known prognostic markers for PFS and OS in myeloma [56].

## 5. Next-Generation Sequencing: Clonotype-Based MRD and Genomic Profiling

The term “NGS” covers two applications that answer different questions and should be kept separate. The first is NGS-based MRD assessment, in which the patient’s clonal immunoglobulin gene rearrangements (IGH, IGK and, less often, IGL) are identified at baseline and then tracked in follow-up samples. Its purpose is to count residual tumor cells, and its performance is expressed as an LOD relative to the number of cells analyzed. The second is genomic profiling by targeted panels, exome or whole-genome sequencing, which looks for somatic mutations, copy-number alterations (CNAs) and structural variants such as translocations. It is used mainly at diagnosis and relapses, for biological characterization, risk stratification and, potentially, treatment selection. Its performance is described in terms of variant allele frequency and sequencing depth. The two approaches differ in workflow, sensitivity and quality requirements. Only the first is currently validated for MRD assessment [16]. Below, we first summarize the genomic background of MM and its assessment at diagnosis (Section 5.1), then discuss clonotype-based MRD (Section 5.2), genomic profiling (Section 5.3) and blood-based NGS approaches (Section 5.4).

### 5.1. Genomic Background and Baseline Risk Assessment

MM pathogenesis begins in the germinal center of a secondary lymph-node follicle, where genomic alterations occur during class-switch recombination and somatic hypermutation of the B-cell receptor [57]. Early-stage MM can be identified in two asymptomatic clinical scenarios: monoclonal gammopathy of undetermined significance (MGUS) and smoldering multiple myeloma (SMM) [58].

Considering age at onset, MM is generally regarded as a disease of the elderly, with only a minority of cases occurring at a younger age. However, the mutational events that pave the way for MM onset and progression appear to occur much earlier, during the second or third decade of life [57].

The main genetic alterations involve two mutually exclusive mechanisms: (I) translocation of oncogenes into the switch region of the IGH locus, caused by aberrant class-switch recombination due to activation-induced deaminase (AID) activity involving CCND1 t(11;14), WHSC1 t(4;14), MAF t(14;16), MAFB t(14;20), and CCND3 t(6;14); (II) hyperdiploid karyotype characterized by multiple trisomies of odd-numbered chromosomes (excluding 13 and 17) [57].

In MGUS these genomic alterations represent the initial events but are not, by themselves, sufficient for progression to MM; additional lesions are required. In general, the greater the number of genomic lesions, the more aggressive the disease. Progressive, more aggressive disease is characterized by major chromosomal-number alterations and AID activity, together with 1q gain or amplification, del(6q), rearrangements or gain of 8q24 (involving the MYC locus), and del(16q) [57].

The evolution from MGUS to SMM remains poorly understood, as MGUS patients are not routinely followed with BM sampling. SMM shares nearly the same clonal genetic abnormalities as MM but often lacks the subclonal driver mutations or microenvironmental changes required for symptomatic progression. In general, the ancestral clone shows greater AID activity, whereas subclones that emerge later are responsible for disease progression and are characterized by APOBEC-related mutational activity and involvement of MYC and *MAPK* genes [57].

Of the three disease stages, MM is the most heterogeneous at the genomic level. Moreover, targeted treatment can promote the outgrowth of subclones that compete with the main clone carrying the actionable mutation [57] (Table 1).

Table 1 is a simplified map of how genomic complexity builds up along the MGUS–SMM–MM continuum. It is not meant as a list of stage-specific markers. Primary events, namely IGH translocations and hyperdiploidy, are already present in MGUS and remain clonal throughout the disease. They define the cytogenetic subgroup, but they are not enough on their own to cause progression. Secondary events, such as gain or amplification of 1q, del(1p), del(13q), del(17p), MYC rearrangements, APOBEC-related mutagenesis and mutations in MAPK-pathway and DNA-repair genes, become more frequent in SMM and especially in active MM. Several of them have prognostic value. 1q gain/amplification, del(1p), del(17p)/TP53 inactivation and t(4;14) are associated with shorter PFS and OS. In contrast, t(11;14) identifies a subgroup that is particularly sensitive to BCL-2 inhibition. The same lesion may be subclonal at one stage and clonal at another, and different subclones may prevail at different time points or in different anatomical sites. Because of this variability, genomic and MRD assessments based on a single sample, taken at a single time point, are always incomplete [57,59].

In routine practice, these lesions are still detected mainly by cytogenetic techniques, and this is unlikely to change in the near future. Conventional karyotyping has a limited yield in MM, because plasma cells grow poorly in culture. Interphase FISH on CD138-purified plasma cells therefore remains the reference method for baseline genetic risk assessment. A standard panel includes t(4;14), t(14;16), t(14;20), t(11;14), del(17p)/TP53 and 1q gain/amplification, often complemented by del(1p) [1,60,61]. Together with serum markers, these abnormalities form the basis of the Revised International Staging System (R-ISS) and its second revision (R2-ISS), which are used for current risk stratification. Some of them also guide treatment, for example the use of venetoclax in t(11;14)-positive disease [1,59]. Cytogenetics and NGS are therefore complementary rather than interchangeable. FISH is fast, widely available and well standardized for the lesions that matter most in the clinic. Sequencing adds mutational information, such as TP53 mutations accompanying del(17p). It also detects bi-allelic events and can find lesions outside the regions covered by the probes [60,61,62].

For this review, the key point is that MRD status should be interpreted together with baseline genetic risk. It does not replace it. A patient with high-risk cytogenetics who becomes MRD-negative still has a different risk of relapse from a standard-risk patient with the same result. In high-risk disease, the durability of MRD negativity seems to matter more than a single negative test [23,24,60,61].

MM is not only the result of random mutagenesis; it also has a genetic component. Germline transmission of risk alleles is well documented, and relatives of patients with MM have a two-fold higher risk of developing a plasma cell dyscrasia [57]. Overall, however, inherited MM remains rare [62]. Heterogeneity is one of the most difficult aspects of MM, both for diagnosis and for understanding its pathogenesis. NGS technologies have played a key role in this area [62]. It is now recognized that only a small number of gene mutations are recurrently represented in NDMM, whereas a large number of mutations are each found in fewer than 10% of NDMM cases. A second layer of complexity comes from subclonal mutations. These are present only in some cells, or only in some anatomical sites, since MM may have an extramedullary component [62].

A third layer of complexity stems from the fact that therapeutic advances over the past 15 years have enabled nearly all patients to achieve a response and more than half to achieve a CR [16]. Nonetheless, most patients eventually relapse, either because of persistent disease below the detection limit of conventional tests or because of the development of treatment resistance. Meeting this challenge requires technologies sensitive enough to detect MRD. It also requires a well-structured follow-up strategy, to find molecular signatures that identify patients at risk of relapse due to resistance to therapy [16,62].

### 5.2. NGS-Based MRD Assessment: Immunoglobulin Clonotype Tracking

The IMWG recommends MRD status, rather than CR, as the preferred outcome measure. When no monoclonal protein can be measured in serum or urine, MRD assessment relies on BM sampling. The most sensitive technologies available are therefore needed, with LODs of 10^−5^ to 10^−6^ (one cell in 10^5^–10^6^ cells), and NGS is one of them [16].

One representative platform is LymphoSIGHT, which relies on amplification of immunoglobulin gene segments (IGH-VDJH, IGH-DJH, or IGK) using locus-specific primers to determine the frequency of different clonotypes [16]. In this setting, the MRD level is expressed as the number of clonotype-specific sequences relative to the total number of cell equivalents analyzed. This differs from the variant allele frequency (VAF) used in mutation profiling, which is the proportion of sequencing reads carrying a given somatic variant [62]. Analysis should be performed early, at diagnosis, and subsequently in all patients with detectable MM clones (>5%) [10]. Moreover, NGS-based MRD cut-offs can further stratify patient outcomes in terms of PFS and OS at different time points [62].

Another example of an NGS-based MRD platform is clonoSEQ, a standardized, FDA-cleared assay based on PCR and NGS. It detects rearrangements of the IGH, IGK and IGL genes, as well as IGH-BCL1 and IGH-BCL2 translocations in BM samples. Each patient sample is assigned a clonotype. The clonotype is compared with a reference database of known Ig rearrangements and given a score, which reflects how likely it is to be found in a healthy repertoire. Clonotypes identified at baseline are then tracked in follow-up samples. The assay is robust, with good repeatability, reproducibility, accuracy and precision [63]. Two practical points deserve emphasis. First, the assay needs a baseline sample with a high tumor burden, usually the diagnostic BM aspirate or an archived specimen, in which the dominant clonotype(s) can be identified. Without it, follow-up samples cannot be interpreted. Second, the nominal LOD of about 10^−6^ is reached only when enough material is analyzed. Since a million diploid cells contain roughly 6.6 µg of DNA, about 20 µg of input DNA (approximately 3 × 10^6^ cell equivalents) is required. When less DNA is available, as in hypocellular or hemodiluted aspirates, the LOD reached in that sample is higher. A negative result should therefore always be reported together with the sensitivity actually reached [63].

NGS is one of the most powerful tools for characterizing this highly heterogeneous disease. Even so, clinical practice has been slow to adopt it. NGS faces several challenges: (I) lack of consensus on how and when to use it; (II) cumbersome sample processing; (III) high cost; (IV) variable availability of the necessary facilities and expertise (laboratory technicians, biologists, bioinformaticians); (V) uncertain reimbursement; (VI) lack of standardization regarding sequencing coverage, methods and variant-calling software [62].

### 5.3. Genomic Profiling by NGS: Mutations, Copy-Number Alterations and Structural Variants

Beyond MRD, a custom targeted NGS panel can detect gene mutations, aneuploidies, segmental CNAs and translocations in a single assay. Traditional approaches, by contrast, need a combination of FISH, karyotyping and SNP arrays. This option could reduce both the cost and the turnaround time of diagnosis [62]. One example is the Unique Molecular Assay (UMA) panel, a customized target enrichment panel designed to identify the most common and clinically relevant gene mutations, CNAs and IGH translocations in MM [64]. The panel gathers the most relevant genomic information on MM in a single experiment. With a dedicated bioinformatic pipeline, it reduces cost, data storage needs and processing time. The UMA panel achieved a positive predictive value (PPV) of 94% and negative predictive value (NPV) of 96.6%, together with a sensitivity of 87.8%, a specificity of 98.4% and an accuracy exceeding 93% [64]. These results are encouraging, but they do not yet justify abandoning conventional cytogenetics. The sensitivity of targeted panels for translocations and subclonal copy-number changes depends on panel design, plasma cell purity and sequencing depth. Costs and turnaround times vary widely between laboratories. Moreover, most risk models and treatment guidelines are still based on FISH-defined categories. For now, targeted NGS is best used together with FISH, not instead of it. Any move towards a single-assay workflow will need prospective validation against established methods [65]. As for availability, the UMA panel was designed and validated in an academic setting, as a custom capture design with its own bioinformatic pipeline. To our knowledge, it is not sold as a commercial in vitro diagnostic kit. A laboratory wishing to use it would have to set it up and validate it in house, as a laboratory-developed test. It would also need the corresponding sequencing platform and bioinformatic expertise [64].

In MM, NGS is best known for clonotype-based MRD tracking. However, genomic profiling at diagnosis could refine conventional NDMM risk stratification, by describing genomic heterogeneity in detail. Sequencing-based methods also allow genome-wide study of the epigenetic status, which is gaining importance in the pathogenesis and prognosis of MM. Examples are bisulfite sequencing for DNA methylation and chromatin profiling (ChIP-seq, ATAC-seq). NGS is not, however, the only option here, since methylation arrays and targeted PCR-based or pyrosequencing assays are also widely used [62].

Genomic profiling could also provide predictive markers to guide treatment selection according to each patient’s genomic profile [62] (Table 2).

Standardization remains a weak point for both applications of NGS. Experimental data suggest that sample transport conditions should be regulated. The technical requirements also differ between the two settings. For clonotype-based MRD, at least 200,000 reads per reaction have been recommended. For genomic profiling, a coverage of 200–500× is suggested for exome studies and >1000× for targeted panels [47,62]. Mean LOD and LOQ values are closely linked to variability in total sample volume, cellularity and viability. The less DNA is available for the reaction, the lower the read depth that can be achieved and, therefore, the sensitivity. Sharing protocols, experience and information is essential to reach consensus and develop harmonized guidelines.

NGS analysis could help make sense of the marked heterogeneity of MM. IRMMa was developed by combining clinical, demographic, genomic and therapeutic data. It provides a genomic classification of NDMM and a model that predicts the individual risk of event-free survival (EFS) and OS [59]. IRMMa shows higher accuracy for EFS and OS than the ISS, R-ISS and R2-ISS staging systems, and can also help identify the optimal treatment based on genomic and clinical features [59].

### 5.4. Blood-Based NGS Approaches

As mentioned above, MRD detection currently relies on BM sampling, a painful procedure that must be repeated during follow-up. Cell-free DNA and circulating tumor cells have therefore attracted interest as promising, minimally invasive tools for follow-up [66].

Starting from 1–5 mL of blood, 80,000 reads per sample can be obtained, sufficient to study B-lineage cells. V(D)J rearrangements detected in cell-free DNA reflect the MM burden across extravascular sites, BM, or extramedullary sites, or all three. V(D)J rearrangements in cfDNA and in leukocyte DNA follow the same trend as the monoclonal protein response. They could therefore allow early prediction of MRD, with less discomfort for patients. These approaches are still investigational. The amount of tumor-derived cfDNA falls sharply when the tumor burden is low, and their sensitivity in the MRD range has not yet been shown to match that of BM-based testing [30].

Several obstacles explain this gap, some technical and some biological. The first is the amount of material. Plasma usually contains only a few nanograms of cfDNA per milliliter, so a standard blood draw yields a few thousand to a few tens of thousands of genome equivalents. This alone puts the theoretical LOD well above the 10^−5^–10^−6^ range reached in BM. In deep remission, the tumor-derived fraction of cfDNA is also extremely small. The amount released depends on tumor turnover and on extramedullary disease, not only on the residual tumor burden. cfDNA is also highly fragmented, mostly into pieces of about 170 base pairs, which makes complete V(D)J rearrangements harder to amplify. Pre-analytical handling matters as well. Relevant factors are the type of collection tube, the time before plasma separation and the degree of leukocyte lysis, which dilutes tumor DNA with genomic DNA from normal cells. When cfDNA is used to look for somatic mutations rather than clonotypes, clonal hematopoiesis adds a further source of false-positive results. Finally, there are no standardized assays or agreed thresholds, and no prospective study has yet shown that cfDNA-based monitoring can take the place of BM-based MRD testing [30,66].

Thanks to its greater sensitivity, NGS can overcome many limitations of other techniques and reveal features otherwise hidden by disease heterogeneity. It can be used on both fresh and stored samples. It can also track clonal evolution, so that resistance-associated variants are identified as soon as they emerge. Overall, clonotype-based NGS is a powerful tool for MRD assessment in BM. Genomic profiling adds biological and prognostic information at diagnosis and at relapse. Blood-based applications are promising but still investigational. However, for logistic and economic reasons, NGS is available only in selected centers, and protocol standardization and data analysis require substantial investment.

## 6. Mass Spectrometry

**Figure 3:**
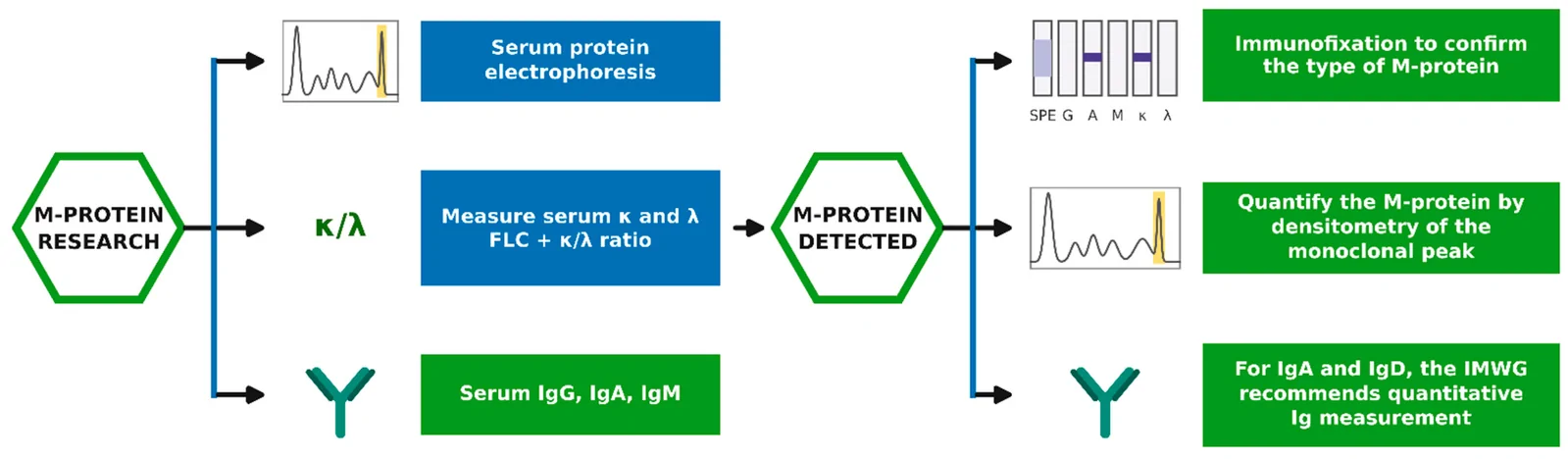
Traditional approaches to M-protein detection. The yellow band marks the monoclonal (M) peak in the electrophoretic and densitometric traces.

MS is a highly sensitive technique for detecting and quantifying monoclonal proteins in the blood of patients with MM. Conventional M-protein assays—serum/urine protein electrophoresis (SPEP/UPEP) and immunofixation electrophoresis (IFE)—have limited sensitivity (approximately 0.2 g/L). They also cannot reliably distinguish residual endogenous M-protein from therapeutic monoclonal antibodies (e.g., daratumumab), which can produce false-positive IFE bands and confound response assessment (Figure 3) [67].

MS overcomes these limitations because it detects M-protein at much lower concentrations than electrophoretic methods. Its analytical sensitivity is far higher than that of conventional blood-based tests (SPEP/IFE) and, in some assays, approaches that of NGS. The two techniques, however, measure different analytes (circulating protein versus tumor-cell DNA), and their results are not directly comparable [68].

MS can also distinguish the patient’s own clonotypic immunoglobulin from therapeutic monoclonal antibodies, based on differences in light-chain sequence or glycosylation. In addition, it can detect oligoclonal or evolving clonal populations that emerge during treatment. Because it is performed on peripheral blood, MS avoids repeated BM aspirations, which are costly and poorly tolerated by patients [69].

This less invasive approach allows frequent longitudinal monitoring with little burden for the patient, and supports an MRD-based therapeutic strategy. MS may predict PFS and OS better than traditional serological methods. Its prognostic value increases over time, particularly when it is combined with BM-based MRD assessment. In some patients, MS detects disease recurrence earlier than conventional methods and gives early warning of relapse. This also applies to patients with non-secretory or oligo-secretory disease, in whom traditional tests may fail. Combining MS with BM and imaging assessments may help define functional cure in MM by identifying patients with sustained MRD negativity [70,71,72].

Several complementary MS workflows have been developed and are at different stages of validation:

Top-down (intact light-chain) methods: analyze intact immunoglobulin light chains using methods such as matrix-assisted laser desorption/ionization time-of-flight mass spectrometry (MALDI-TOF), Mass-Fix and EXENT^®^. These methods do not require a baseline sample and are suitable for routine clinical use. MALDI-TOF is a relatively rapid, semi-quantitative platform for immunoglobulin isotyping and M-protein detection applied directly to serum. It has been used to track M-protein in peripheral blood as a surrogate for BM MRD. Its concordance with flow cytometry-based MRD was reasonable, although its analytical sensitivity is lower than that of liquid chromatography-MS (LC-MS) approaches. MALDI-TOF analysis of peripheral blood has also been compared with BM-based flow cytometry for MRD tracking in patients with MM [73].

Bottom-up (clonotypic peptide) methods: track unique peptide sequences from the patient’s monoclonal protein (e.g., EasyM, M-InSight). These are more sensitive but require a baseline sample for tracking [74].

These targeted LC-MS/MS assays first sequence the patient’s unique clonotypic immunoglobulin variable-region peptide in a diagnostic sample. This peptide is then used to design a targeted, highly sensitive LC-MS/MS assay that quantifies it in serial serum samples. In one study, serial serum samples from patients with MM were analyzed with the EasyM protocol and compared with immunofixation electrophoresis, multiparameter flow cytometry and NGF. EasyM tracked M-protein dynamics with greater sensitivity than standard serology. Similar clonotypic peptide-based workflows have been developed using immunoglobulin variable-region sequences identified from diagnostic BM or serum [74,75].

Finally, immunoglobulin-enrichment LC-MS methods (e.g., M-InSight, miRAMM-type assays) enrich serum immunoglobulins (often via nanobody- or bead-based capture) before LC-MS analysis. Their LODs approach 10^−6^ and in some cohorts their performance is close to that of BM-based NGS [33,69,76].

In a pooled analysis of patients treated with carfilzomib-based regimens, MRD was assessed in BM by NGS and in peripheral blood using two MS methods, MALDI-TOF (EXENT) and the more sensitive LC-MS. Negativity on the more sensitive LC-MS assay identified patients with long-term treatment responses. Patients negative on both LC-MS and NGS MRD testing at the 10^−6^ threshold had a five-year PFS rate of 89% [71].

A study based on data from the IFM-2009 clinical trial compared blood-based targeted MS with BM NGS for response assessment. MS-MRD on frozen serum was evaluated against routine M-protein diagnostics and NGS-based MRD [68].

A recent systematic review included studies published between 2021 and 2025. The cohorts ranged from 26 to 458 patients with newly diagnosed, transplant-eligible, high-risk and smoldering myeloma. The authors concluded that MS-based proteomic biomarkers are robust enough to provide independent prognostic information on the likelihood of disease progression. They also concluded that MS-based MRD detection is superior in sensitivity and prognostic value to standard serologic techniques, such as immunofixation electrophoresis, and to NGS [72]. These conclusions, however, rest on heterogeneous and largely retrospective cohorts, analyzed with different assays and thresholds, and should be interpreted with caution.

Targeted MS assays have also been used for serial monitoring of MRD status during treatment. They capture the kinetics of M-protein decline and early molecular relapse, which a single BM sample taken at one time point would miss. This could help adapt treatment intensity or duration [77].

MS also has several limitations for MRD monitoring in MM. Standardized, harmonized assay protocols and reporting thresholds are still lacking across centers. MS requires a baseline (diagnostic) sample to define the patient-specific clonotypic peptide, limiting its use in patients without an archived diagnostic specimen. Blood-based MS reflects circulating M-protein dynamics, which may not always match BM tumor burden (e.g., in light-chain or non-secretory myeloma). Moreover, clinical-grade, regulatory-approved assays remain limited, and most evidence still derives from research-use platforms. Finally, a universally accepted sensitivity threshold, analogous to the 10^−5^/10^−6^ convention used for NGF/NGS, has yet to be established [78,79].

Ongoing studies have several aims. The first is to harmonize MS-MRD assay design and reporting. Others are to validate sensitivity thresholds prospectively against hard clinical endpoints (PFS/OS) and to extend use to light-chain and oligo-secretory disease. MS-based blood monitoring is also being included in adaptive treatment trials, in which MRD status guides de-escalation or intensification of therapy [69,80,81].

Because of their close interdependence with the surrounding microenvironment, clonal plasma cells can expand and produce large amounts of monoclonal component. This may be an intact Ig, an FLC, both or neither [82,83].

A biomarker is an objectively measured characteristic used to evaluate normal biological processes, pathogenic mechanisms, or therapeutic responses [84].

In patients with normal renal function, the presence of FLCs in the circulation is directly related to B-cell activity. FLCs are also bioactive molecules that mediate cellular interactions, including activation of mast cells, neutrophils, and B cells. They have been shown to have enzymatic activity (anti-angiogenic, proteolytic and prothrombinase activity) and to participate in complement activation [85].

Because FLCs are produced by a single B-cell clone following malignant proliferation, they are routinely evaluated in clinical practice for the diagnosis and monitoring of plasma cell dyscrasias, including MM [7].

Even with an intact monoclonal immunoglobulin, excess FLC production frequently occurs and may become the predominant or only detectable marker at progression. Intraclonal heterogeneity and clonal evolution are general features associated with disease progression and treatment resistance in myeloma. The clinical expression of this clonal heterogeneity is LCE, defined as an increase in the involved FLC without a corresponding increase in the original monoclonal intact Ig [10].

For this reason, FLC measurement during follow-up of MM patients is essential to avoid missing LCE, and MS may provide an important tool in this setting.

A “double-negative” composite endpoint, which combines blood-based MS with marrow-based molecular methods, looks particularly promising. It may help identify patients with the deepest and most durable remissions [71]. This composite endpoint, however, has been studied mainly in retrospective or post hoc analyses. It has not been validated in multicenter prospective trials, and it cannot yet be used as a standard in routine practice; for now, its place is within clinical trials.

MS is a sensitive, minimally invasive, blood-based complement to BM flow cytometry and sequencing for MRD assessment in MM. MS-based assays, such as EasyM, M-InSight and MALDI-TOF/EXENT-based platforms, track a patient-specific clonotypic immunoglobulin peptide. They allow frequent monitoring with little burden for the patient, and add independent prognostic information to conventional serology. For now, though, MS should be seen as a complementary monitoring tool, not as an established substitute for BM-based MRD assessment. Some important gaps remain. No positivity threshold has been agreed internationally, and, with few exceptions, the reagents and assays in use are labeled for research use only. For these reasons MS cannot, at present, replace NGF or NGS as the reference method for defining MRD-based primary endpoints in clinical trials, or for MRD assessment under the IMWG criteria [33,78,79].

In 2026, the Spanish Myeloma Group proposed a new definition of CR, comparing traditional IMWG criteria with the EXENT method. The study showed MS to be a more reliable indicator of response depth than morphology alone. This approach introduces an “MS-CR” category. It suggests that MS could complement BM-based methods in assessing treatment effectiveness, although prospective validation is still needed [86].

Broader clinical adoption will depend on assay standardization, prospective validation of sensitivity thresholds, and regulatory qualification. The IMWG has set up a dedicated Mass Spectrometry Committee to evaluate and standardize the use of MS in the diagnosis and monitoring of MM. The committee works closely with the MRD Committee on clinical testing guidelines [33].

## 7. Discussion

Over the past decade, the treatment of MM has changed rapidly, and patient outcomes have improved markedly. New agents and sequential treatment phases have led to deeper responses and longer PFS. MRD assessment has had a major clinical impact. It has redefined the concepts of “remission” and “prognosis,” and it is changing drug development and personalized treatment strategies [87,88,89,90,91].

Despite high CR rates, most patients eventually relapse. This is particularly true for those with high-risk (HR) cytogenetic abnormalities, even when their initial response rates are similar to those of standard-risk (SR) patients. More sensitive response assessment is therefore needed, together with new criteria able to identify deeper responses than current definitions. MRD is increasingly seen as a more informative measure of response depth. It helps identify patients at higher risk of relapse and could potentially guide response-adapted treatment strategies [92]. Traditional response criteria based on serum and urine monoclonal protein, BM plasma cell percentage, and serum free light chain assays are insufficient to detect low levels of residual disease [93].

In clinical trials, higher CR rates and longer survival have led to the use of MRD negativity, rather than conventional CR, as a more accurate measure of response depth. MRD negativity is also used as a surrogate endpoint for PFS and OS. MRD platforms are increasingly sensitive and standardized, and guidelines for MRD measurement and reporting have been harmonized. This has reduced heterogeneity in MRD analysis. It has also helped define the best methods, the assessment time points and how results should be interpreted and reported [81,94].

MRD can be assessed with BM-based, peripheral blood-based or imaging-based techniques. Imaging-based approaches are not reviewed in detail here, although, as discussed below, they are essential for a correct interpretation of MRD results [95].

Three techniques are currently used—NGF, NGS and, more recently, blood-based MS. Table 3 compares their main features, using the same categories for the three methods [16,28,33,63,69]. Note that the analytical sensitivity of cell-based assays and that of MS cannot be directly compared. The first is expressed as a fraction of nucleated cells, the second as a protein concentration.

**Table 3:** Comparison of NGF, NGS and MS for MRD assessment in MM. Values are indicative and depend on the specific assay, sample quality and laboratory setting.

	NGF	NGS (Clonotype-Based)	MS
Analyte	Aberrant plasma cells (immunophenotype)	Clonotypic IGH/IGK/IGL rearrangements (DNA)	Clonotypic M-protein (intact light chain or clonotypic peptides)
Sample	BM aspirate (PB for CTCs: investigational)	BM aspirate (PB/cfDNA: investigational)	PB (serum)
Baseline sample required	No	Yes (to identify the clonotype)	• Top-down (intact light chain): no • Bottom-up (clonotypic peptide): yes
Pre-analytical requirements	• Fresh BM aspirate (first pull; EDTA or heparin), kept at room temperature and processed ideally within 24 h • ≥10^7^ nucleated cells to be acquired • Frozen/thawed material not suitable	• BM aspirate (first pull; EDTA) • DNA is stable: samples can be shipped, frozen or stored as extracted DNA • ≥20 µg DNA needed for an LOD of 10^−6^ • Baseline sample with high tumor content	• Serum (standard clot tube) after routine centrifugation; stable when frozen • Bottom-up: stored diagnostic serum needed to define the clonotypic peptide • Information on therapeutic mAbs needed for interpretation
Applicability	~100% of patients	~90% (clonotype identifiable at baseline)	Secretory disease; limited in oligo- and non-secretory MM
Analytical sensitivity (LOD)	~2 × 10^−6^ when ≥10^7^ cells are acquired (EuroFlow); 10^−5^ with conventional MFC	~10^−6^ with ≥20 µg DNA (~3 × 10^6^ cell equivalents)	Expressed as concentration, not as a cell fraction: • Intact-protein MS: ~0.1 g/L • Bottom-up MS: ~0.001 g/L
Turnaround time	Same day (a few hours)	Up to ~7 days (often centralized)	• Intact-protein MS: ~20 s per acquisition (excluding sample preparation) • Bottom-up MS: ~2 h per run, plus baseline peptide identification
Relative cost (setting-dependent)	+++	+++++	• Intact-protein MS: +++ • Bottom-up MS: ++++
Standardization/regulatory status	EuroFlow standard operating procedures; IMWG-endorsed	IMWG-endorsed; FDA-cleared commercial assay available (clonoSEQ)	Mostly laboratory-developed or research-use assays; harmonization ongoing (IMWG Mass Spectrometry Committee)
Clinical validation	Prognostic value shown in prospective trials; accepted for MRD endpoints	Prognostic value shown in prospective trials; accepted for MRD endpoints	Prognostic value shown in trial cohorts, mostly retrospective; not yet included in IMWG response criteria
Main advantages	• High sensitivity • No baseline sample needed • Fast turnaround • Wide availability • High reproducibility • Sample quality (hemodilution) can be assessed	• Highest standardized sensitivity • Small sample volume • Can be performed on stored samples	• Minimally invasive • Suitable for frequent serial monitoring • High specificity; distinguishes therapeutic mAbs • High throughput
Main limitations	• Invasive • Large volume needed • Fresh sample needed • Expertise required • Sampling bias (patchy infiltration; extramedullary disease missed)	• Invasive • Baseline sample needed • Hemodilution not directly assessable • No phenotypic information • Longer turnaround • Limited availability • Sampling bias	• Measures protein, not cells • M-protein half-life delays response assessment • Limited in oligo-/non-secretory disease • Bottom-up methods need baseline sequence data and expertise • Limited standardization
Current role	Reference BM-based method	Reference BM-based method	Complementary blood-based monitoring

Relative cost (+++, ++++, +++++) is graded on a scale from + (lowest) to +++++ (highest); it depends on the setting.

NGF has several strengths. Results are available on the same day and the method is widely available in laboratories. It needs neither a baseline sample nor the prior identification of a tumor-specific marker. It has, however, two consistent limitations. Samples must be processed fresh, since frozen/thawed samples may lose sensitivity, and EuroFlow-trained staff are needed to ensure full standardization [96].

NGS-based MRD is highly sensitive and allows the dominant clone to be tracked over time. Combined with genomic profiling, sequencing can also reveal clonal evolution and emerging drug-resistance mutations, and so help adjust treatment. However, high costs, lack of standardization, slow processing and complex data analysis still limit its routine use [97].

At present, the choice between NGS and NGF for MRD evaluation in MM largely depends on local availability and on whether a baseline BM sample is available. Ideally, the two techniques should be combined, to assess the molecular and cellular features of MM at the same time [41,98].

BM-based NGF and NGS, with an LOD of at least 10^−5^, remain the current standard for MRD testing in MM. However, BM aspirations must be repeated, and false-negative results may occur because of patchy marrow involvement or isolated extramedullary disease. For these reasons, peripheral blood-based MRD tools have become increasingly attractive. MS-based approaches are among the most promising of these, for relatively non-invasive, dynamic MRD monitoring. MS needs only a peripheral blood draw. It is suitable for frequent, longitudinal monitoring and avoids sampling errors due to focal or patchy marrow infiltration. Nevertheless, it detects circulating protein and not the malignant cells themselves, so its results are not strictly equivalent to those of cell-based methods. MS is also less standardized, is not yet part of the IMWG response criteria and is less informative in oligo-secretory or non-secretory disease [99,100].

MS is the fastest-growing complementary approach. It allows frequent, minimally invasive monitoring on blood, and in some studies it has detected molecular relapse earlier than conventional serological monitoring. It has not, however, replaced the marrow-based gold standards in formal response criteria [101].

Few studies have compared the three techniques directly in the relapse patterns shown in Figure 2, but their analytical principles allow some reasonable predictions. NGF and NGS detect residual plasma cells whatever these cells secrete, so they should perform in much the same way in all three scenarios. NGF recognizes aberrant plasma cells by their immunophenotype and, in the second EuroFlow tube, by cytoplasmic light-chain restriction, which is preserved in clones that no longer produce the heavy chain. NGS follows the clonotype identified at baseline. Escaping subclones usually derive from the same ancestral clone and carry the same rearrangement. If heavy-chain loss is caused by deletion or rearrangement of the IGH locus, however, a strategy limited to IGH might underestimate the escaping clone. Tracking IGK or IGL as well reduces this risk. MS is the technique most affected by the pattern of secretion. Relapses with both intact immunoglobulin and FLCs (type 1) or with intact immunoglobulin only (type 3) can be picked up by intact light-chain and clonotypic peptide methods alike. In light chain escape (type 2), the assay has to target the light chain, either its intact mass or peptides from its variable region. Methods based on heavy-chain peptides will miss it. Here the short half-life of FLCs is an advantage, since changes show up in serum within days, whereas the long half-life of intact IgG delays the recognition of both response and relapse in types 1 and 3. Lastly, when the escaping clone becomes oligo- or non-secretory, blood-based MS loses most of its usefulness, and BM-based NGF or NGS, together with imaging, are still needed [10,33,69].

In 8–21% of patients, blood-based tests do not detect residual disease that is found in the BM [56]. These cases are usually called false negatives, but the term can be misleading. Peripheral blood and BM are different compartments. The amount of disease found in each depends on three factors: tumor burden, distribution of the disease between the marrow and other intramedullary or extramedullary sites, and disease biology. Examples of biological factors are the tendency of plasma cells to circulate and, for protein-based tests, the half-life of the M-protein. A discordant result may therefore reflect the real distribution of the disease, and not a technical failure of either test. For this reason, different MRD techniques are increasingly used together. The aim is to obtain more sensitive results and, potentially, to use MRD to guide tailored therapy.

More generally, MRD testing has intrinsic limits in a disease as heterogeneous as MM. Marrow infiltration is often patchy. Multi-region sequencing has shown that focal lesions may contain subclones, including high-risk ones, that are absent from the iliac crest aspirate [102]. A single BM sample can therefore underestimate the residual tumor burden, and an MRD-negative marrow does not exclude persistent focal or extramedullary disease. Functional imaging covers part of this blind spot. The IMWG recommends PET/CT for assessing metabolically active disease. Its response criteria include an “imaging plus MRD-negative’ category. Besides marrow negativity, this requires that every area of increased tracer uptake found at baseline disappears, or falls below the level of the mediastinal blood pool or of the surrounding normal tissue [16,103]. In prospective studies, normalization of PET/CT before maintenance therapy was an independent predictor of PFS and OS [104]. Bone marrow, peripheral blood and imaging therefore provide different and partly independent views of the same disease. None of them is sufficient on its own. Discordant findings should be interpreted in their clinical context, and not automatically attributed to a technical error.

Non-secretory and extramedullary MM deserve a separate comment. In these settings a blood-based test would be most useful, but current tools are weakest. In non-secretory disease, cell-based methods do not depend on what the clone secretes. NGF recognizes aberrant plasma cells by their phenotype, and NGS follows the clonotypic rearrangement. Both can therefore be used on BM, provided that a baseline sample is available for NGS. Serum FLCs can be followed in the many patients whose disease is in fact oligo-secretory [8]. MS, which measures circulating M-protein, adds little when no monoclonal protein can be detected. Extramedullary disease carries a poor prognosis [105] and raises the opposite problem. Soft-tissue plasmacytomas may persist or progress while the marrow is MRD-negative, and their response is assessed mainly by PET/CT [16,103]. In both situations, circulating tumor-derived nucleic acids are an attractive option. They are released from every disease site, not only from the aspirated marrow. In paired BM and plasma samples, about half of the mutations identified were detectable in plasma. Mutations found only in plasma were much more frequent in relapsed/refractory than in newly diagnosed patients. This is consistent with spatial heterogeneity and with disease outside the sampled marrow [106]. In a patient with oligo-secretory, extramedullary MM, rising levels of a mutation tracked in plasma cfDNA followed the progression of extramedullary lesions on PET/CT while the marrow disease was responding [107]. Extracellular vesicles (EVs) are another possible source of tumor-derived material. Circulating exosomal microRNAs were independent predictors of PFS and OS in newly diagnosed MM [108], and EV-derived RNA is being explored for diagnosis and monitoring [109]. These data are encouraging, but they come from small series and single cases. The limitations of cfDNA discussed in Section 5.4 apply here as well. For EVs, isolation methods and pre-analytical handling are not standardized, and tumor-derived vesicles are hard to distinguish from the much more abundant platelet-derived ones [66,109]. Neither approach has been validated for MRD assessment. For now, MRD evaluation in non-secretory and extramedullary MM still relies on BM-based NGF or NGS, combined with functional imaging.

Sensitive MRD detection is useful in many aspects of MM management. It supports risk stratification, the definition of prognosis and the early prediction of relapse, and it is increasingly used as a clinical trial endpoint. MRD is also emerging as a tool to guide treatment intensity and duration [110].

These uses rest on different levels of evidence and should be kept distinct. First, MRD is a prognostic marker: the association between MRD negativity and longer PFS and OS is consistent across trials, treatment settings and techniques [19,20,21,22,23,24]. Second, MRD is a regulatory endpoint. Based on large patient-level meta-analyses, the FDA has accepted MRD negativity as an endpoint to support accelerated approval. It has since proposed MRD and CR as primary endpoints in MM trials [17,18]. This applies to trial populations and does not, by itself, show that changing therapy according to an individual patient’s MRD result improves outcomes. Third, the use of MRD to guide treatment decisions is still under investigation. Examples are stopping maintenance in patients with sustained negativity, or intensifying treatment in those who remain positive. Studies such as MRD2STOP support the feasibility of MRD-guided treatment discontinuation [26], and several prospective MRD-adapted trials are ongoing. Until their results are available, changes to therapy based on MRD should preferably be made within clinical trials [99,101,110].

Artificial intelligence (AI) is also playing a growing role in this field. It is already used in clinical practice through automated platforms and algorithms that produce precise and accurate results [111], for example in NGS analysis pipelines [112]. The next step is a wider use of machine learning and AI to support clinical decisions and improve risk stratification. Examples are the IRMMa score and the Connector R-package for MRD assessment [15,112,113].

AI may reduce manual workload, turnaround time and variability within and between laboratories and operators. It may also improve the standardization of NGF, NGS and MS, as shown by the CCADDAS (Clustering and Classification of All events, Dimensionality reduction, Down-sampling, and Aberrancy Scaling) pipeline [114]. AI can also integrate and analyze large amounts of heterogeneous data, thereby helping researchers and clinicians find features that might otherwise be missed. However, large datasets alone are not enough to support clinical decisions. Algorithms, machine-learning models and AI applications in general must be properly standardized and validated [112,113,115]. These developments confirm the growing value of MRD in evaluating new therapies and in personalizing treatment. Even so, fully integrating MRD data into routine clinical management is still a challenge. Methods still differ in measurement, sensitivity thresholds, reporting and clinical interpretation of results. Many ongoing clinical trials are exploring the role of MRD in guiding treatment and should clarify this point [116].

Finding the best approach to MRD assessment in routine practice could help in several ways. It could support risk-adapted, personalized treatment, define suitable monitoring schedules for MM patients and improve long-term disease management while limiting treatment-related toxicity [58].

A key priority for the future is to develop and validate methods for detecting and monitoring MRD in peripheral blood. These non-invasive approaches would allow more frequent testing without repeated bone marrow sampling, which is more comfortable for patients. The role of AI also needs further study, as it may help in the interpretation of MRD results. Current evidence suggests that combining two different analytical methods may be the best way to identify patients with residual disease accurately. MRD results should also be read together with baseline genetic risk and imaging. Multicenter prospective trials have yet to show whether a composite marrow-plus-blood endpoint should be used to guide treatment. Until then, it should be confined to clinical studies. Likewise, MS cannot replace NGF or NGS as the reference for MRD-based endpoints until a common positivity threshold and clinical-grade reagents are available.

## Figures and Tables

**Table 1:** Main genomic features of MGUS, SMM and MM and their biological or clinical significance.

Disease Stage	Main Genomic Features	Biological/Clinical Significance	Ref.
MGUS	• Primary IGH translocations: t(11;14) CCND1, t(4;14) NSD2 (WHSC1)/FGFR3, t(14;16) MAF, t(14;20) MAFB, t(6;14) CCND3 • Hyperdiploidy (trisomies of odd-numbered chromosomes)	Initiating, clonal events that define the cytogenetic subgroup; retained at progression but not sufficient, on their own, for progression	[57]
SMM	• CNAs: del(13q), del(6q), del(8p), del(16q), amp(1q) • APOBEC mutational activity • MYC translocations or other MYC abnormalities; mutations in MAPK-pathway or DNA-repair genes; t(4;14)	Accumulation of secondary, often subclonal, events; greater genomic complexity and APOBEC activity are associated with a higher risk of progression to active MM	[57]
MM	• Recurrent driver mutations: NRAS, KRAS, BRAF, DIS3, EGR1, LTB, TENT5C, TRAF3 • CNAs: del(1p), amp(1q), del(13q), del(14q), del(16q) • Co-occurring patterns: t(4;14) with del(13q), CNAs, DIS3 and FGFR3 mutations; t(11;14) with CCND1 and IRF4 mutations, or with bi-allelic TP53 inactivation	Highest genomic heterogeneity, with branching subclonal evolution; RAS/MAPK mutations are potentially actionable; amp(1q), del(1p) and bi-allelic TP53 inactivation identify high-risk disease; co-occurrence patterns define the genomic subgroups used in individualized risk models (e.g., IRMMa)	[57,59]

**Table 2:** Genomic markers reported to be associated with response or resistance to the main drug classes used in MM. Most associations come from retrospective or translational studies and are not yet used to guide treatment in routine practice.

Drug Class	Genomic Marker	Reported Association	Ref.
All classes (general high-risk features )	Bi-allelic inactivation of tumor suppressor genes (e.g., TP53); amp(1q); APOBEC mutational signature; del(17p)	High-risk, chemoresistant disease, with shorter PFS and OS regardless of therapy	[57]
IMiDs (lenalidomide, pomalidomide)	CRBN, IKZF1 and IKZF3 mutations (cereblon pathway)	Acquired resistance to IMiDs; CRBN hotspot mutations described in lenalidomide-refractory disease	[62]
IMiDs	EGR1 and IRF4 mutations	Favorable outcome in patients treated with IMiD-containing regimens	[62]
IMiDs	ZFHX4 mutations	Unfavorable outcome in patients treated with IMiD-containing regimens	[62]
IMiDs	IGL (immunoglobulin lambda locus) translocations	Adverse prognosis and apparent lack of benefit from IMiDs	[62]
Proteasome inhibitors (bortezomib, carfilzomib)	PSMB5, PSMB8, PSMB9, PSMD1 and PSMG2 mutations (proteasome subunits)	Resistance to PIs, particularly bortezomib	[62]
Proteasome inhibitors	t(4;14); TRAF3 deletion	Good response to bortezomib	[62]
BCL-2 inhibitors (venetoclax)	High BCL2/MCL1 and BCL2/BCL-XL RNA ratios	Predictive of response to venetoclax	[62]
BCL-2 inhibitors	BCL2 mutations; MCL1 up-regulation	Resistance to venetoclax	[62]
BRAF inhibitors	BRAF V600E mutation	Actionable target, but resistance to BRAF inhibitors develops rapidly	[62]
SINE (selinexor)	XPO1 mutations	Resistance to selinexor	[62]

## Data Availability

No new data were created or analyzed in this study. Data sharing is not applicable to this article.

## Abbreviations

Abbreviation	Definition
AI	artificial intelligence
AID	activation-induced deaminase
amp	amplification
APC	allophycocyanin
aPC	aberrant plasma cell
APOBEC	apolipoprotein B mRNA editing enzyme, catalytic polypeptide-like
ASCT	autologous stem cell transplantation
ATAC-seq	assay for transposase-accessible chromatin with sequencing
BCMA	B-cell maturation antigen
BCR	B-cell receptor
BM	bone marrow
BV	Brilliant Violet
CAR	chimeric antigen receptor
CCADDAS	Clustering and Classification of All events, Dimensionality reduction, Down-sampling and Aberrancy Scaling
CD	cluster of differentiation
CDR	complementarity-determining region
cfDNA	cell-free DNA
ChIP-seq	chromatin immunoprecipitation sequencing
CNA	copy number alteration
CR	complete response
CTC	circulating tumor cell
cy	cytoplasmic
del	deletion
DGM	density gradient medium
EFS	event-free survival
EHA	European Hematology Association
EMN	European Myeloma Network
EV	extracellular vesicle
FDA	US Food and Drug Administration
FISH	fluorescence in situ hybridization
FITC	fluorescein isothiocyanate
FLC	free light chain
HR	high risk
IFE	immunofixation electrophoresis
Ig	immunoglobulin
IGH	immunoglobulin heavy chain locus
IGK	immunoglobulin kappa light chain locus
IGL	immunoglobulin lambda light chain locus
IMiD	immunomodulatory drug
IMWG	International Myeloma Working Group
IRMMa	Individualized Risk Model for Multiple Myeloma
ISS	International Staging System
R-ISS/R2-ISS	Revised/Second Revision of the International Staging System
LC-MS	liquid chromatography–mass spectrometry
LC-MS/MS	liquid chromatography–tandem mass spectrometry
LCE	light chain escape
LOD	limit of detection
LOQ	limit of quantification
mAb	monoclonal antibody
MALDI-TOF	matrix-assisted laser desorption/ionization time-of-flight (mass spectrometry)
MAPK	mitogen-activated protein kinase
ME	multiepitope
MFC	multiparameter flow cytometry
MGUS	monoclonal gammopathy of undetermined significance
miRAMM	monoclonal immunoglobulin rapid accurate mass measurement
MM	multiple myeloma
M-protein	monoclonal protein
MRD	measurable (minimal) residual disease
MRI	magnetic resonance imaging
MS	mass spectrometry
NDMM	newly diagnosed multiple myeloma
NGF	next-generation flow (cytometry)
NGS	next-generation sequencing
NPV	negative predictive value
OS	overall survival
PB	peripheral blood
PC	plasma cell
PCR	polymerase chain reaction
PE	phycoerythrin
PerCP	peridinin-chlorophyll protein
PET/CT	positron emission tomography/computed tomography
PFS	progression-free survival
PI	proteasome inhibitor
PPV	positive predictive value
RRMM	relapsed/refractory multiple myeloma
RUO	research use only
SINE	selective inhibitor of nuclear export
SMM	smoldering multiple myeloma
SNP	single-nucleotide polymorphism
SPEP	serum protein electrophoresis
SR	standard risk
t	translocation
UMA	Unique Molecular Assay
UPEP	urine protein electrophoresis
VAF	variant allele frequency
V(D)J	variable–diversity–joining (immunoglobulin gene segments)
WBC	white blood cells
κ/λ	kappa/lambda (immunoglobulin light chains)
Note: gene and locus symbols (e.g., CCND1, WHSC1, MAF, MAFB, CCND3, MYC, NRAS, KRAS, BRAF, DIS3, EGR1, LTB, TENT5C, NSD2, TRAF3, TP53, IRF4, FGFR3, CRBN, IKZF1, IKZF3, PSMB5, XPO1, ZFHX4, BCL2, MCL1) follow standard HGNC nomenclature and are not expanded.	
